# Effect of Skin-to-Skin Care on the Day of Birth on Skin Colonization in Preterm Infants: A Pre- and Post-Implementation Study

**DOI:** 10.3390/children11121506

**Published:** 2024-12-10

**Authors:** Poorva Deshpande, Nosheen Akhtar, Maura Mansur, Allison McGeer, Vibhuti Shah

**Affiliations:** 1 Department of Paediatrics, Mount Sinai Hospital, Toronto, ON M5G 1X5, Canada; poorva.deshpande@sinaihealth.ca (P.D.); nosheen.akhtar@sickkids.ca (N.A.); maura.mansur@thp.ca (M.M.); 2 Department of Microbiology, Mount Sinai Hospital, Toronto, ON M5G 1X5, Canada

**Keywords:** preterm, skin-to-skin, skin colonization

## Abstract

Background/Objectives: Maternal skin-to-skin contact (MSSC) in neonates has been shown to reduce nosocomial infections. In preterm infants, exposure to maternal skin commensals within the first 24 h may prevent colonization by hospital-acquired pathogens. However, the impact of early MSSC on skin colonization in preterm infants is unknown. Our aim was to compare skin colonization patterns on days 2, 3, and 7 of life in preterm infants (28^0/7^ to 31^6/7^ weeks gestational age) who received MSSC within the first 24 h from birth with those who did not. The primary outcome was the rate of skin colonization with bacterial pathogens. The secondary outcome was the rate of *Staphylococcus aureus* colonization. Methods: This prospective pre- and post-implementation study was conducted at Mount Sinai Hospital, Toronto. Skin swabs were obtained at 24–36 h, 48–72 h, and day 7 of life. Infant mouth and rectal swabs were collected on day 7. Maternal nasal–rectal swabs were obtained at any time from recruitment to day 7. Results: Twenty-seven infants were included in the pre-implementation group and seventeen were included in the post-implementation group, respectively. Post-implementation infants received an increased duration of SSC during the first week. No differences in colonization with pathogens vs. commensals or *Staphylococcus aureus* colonization were observed between groups at any time point. Skin was fully colonized in both groups by day 7. Conclusions: No differences in skin colonization patterns were identified in the first week of life for preterm infants receiving early MSSC. Larger studies with longitudinal data are needed to further evaluate the impact of MSSC on skin colonization.

## 1. Introduction

Bacterial colonization of neonatal skin is inevitable and mostly begins after exposure to the extra-uterine environment, although there have been some recent suggestions of colonization starting in utero [1,2]. While term infants are typically colonized with maternal flora [3], preterm infants in neonatal intensive care units (NICUs) are more likely to be colonized by potentially pathogenic hospital-acquired organisms [4]. This colonization pattern, particularly with antibiotic-resistant strains, may increase the risk of nosocomial infections (NI) [5]. Nosocomial infections pose a significant challenge for preterm infants in NICUs [6]. The incidence of NI in infants born at <33 weeks is approximately 3.2 infections per 1000 patient days, with Gram-positive organisms, specifically coagulase-negative staphylococci (CONS), being the most common pathogens [6,7]. Factors associated with increased NI risk include lower birth weight, younger gestational age, use of central venous catheters, parenteral nutrition, and mechanical ventilation.

Maternal skin-to-skin contact (MSSC), also referred to as Kangaroo Mother Care (KMC), has shown numerous benefits, including improved breastfeeding outcomes, better cardiorespiratory stability, enhanced neurodevelopment, and reduced procedural pain [8,9]. Recent studies have suggested that MSSC may also play a role in reducing the incidence of NI in preterm infants [10,11,12]. The mechanism behind this potential benefit is postulated to involve colonization with maternal commensal organisms, which may competitively inhibit the growth of hospital-acquired pathogens [13]. However, evidence regarding the impact of early MSSC on skin colonization patterns and subsequent NI risk in preterm infants remains limited. Therefore, the aim of this study was to investigate the effect of early MSSC on skin colonization patterns in preterm infants during the first week of life. Our specific primary objective was to measure the rate of colonization (skin and oral/rectal) with bacterial pathogens at between 24 and 36 h, 48 and 72 h, and on day 7 of life in clinically stable preterm infants without risk factors for early- onset sepsis other than prematurity, born between 28^0/7^ and 31^6/7^ weeks gestational age (GA). The secondary objective was to determine the rate of colonization with different subtypes of Staphylococcus aureus (*S. aureus*). We hypothesized that early MSSC is associated with skin colonization with fewer pathogenic bacteria.

## 2. Materials and Methods

### 2.1. Study Design and Setting

This prospective pre- and post-implementation study was conducted in the NICU of Mount Sinai Hospital, Toronto. The implementation of early MSSC for stable preterm infants within the first 24 h of life began in April 2013.

### 2.2. Inclusion and Exclusion Criteria

Preterm inborn infants born between 28^0/7^ and 31^6/7^ weeks GA were eligible for inclusion. Infants with prolonged rupture of membranes >18 h, suspected maternal chorioamnionitis (defined as clinical suspicion as per the obstetrical team such as intrapartum fever, with or without leucocytosis or malodorous or discolored vaginal discharge), maternal Group B streptococcal colonization, maternal active herpes simplex virus or human immunodeficiency virus infection, clinical instability of the infant (defined as receiving >50% supplemental oxygen on mechanical ventilation or non-invasive ventilation, or inotropic medications), the presence of a chest tube, or known congenital or genetic abnormalities were excluded.

### 2.3. Study Interventions

Parental consent was obtained either prior to delivery or on the first day after birth. In both groups, infants were stabilized at birth by the attending medical team, which included provision for respiratory support as needed, placement of intravenous or umbilical lines catheters, and administration of drugs. In the pre-implementation period, there was no guideline for provision of MSSC, and therefore it was performed at the discretion of the attending medical and bedside nursing team.

In the post-implementation group, infants received early MSSC, defined as at any time within the first 24 h of birth for a minimum duration of 30 min (no maximum time limit). For MSSC, the mother was seated on a reclining chair or a wheelchair, if there were mobility issues. The infant was transferred from the resuscitaire or incubator to the mother’s chest by the nursing staff and placed prone between the mother’s breasts. The infant was dressed in a diaper and a hat and the infant’s back was covered with a receiving blanket folded into fourths. The mother was encouraged to place her hands lightly on the receiving blanket covering the infant’s back but advised not to stroke the infant. The infant’s heart rate, respiratory rate, and SpO_2_ (oxygen saturations) were continuously monitored on the bedside physiological monitor and recorded every 5 min from the monitors. Temperature was obtained by using an electronic thermometer (FILAC 3000^TM^, Covidien) at the beginning of MSSC, recorded every 10 min, and at the end of MSSC. If the temperature dropped to 36.2° Celsius (C) or lower, the infant was covered with extra warm blankets. If, after a period of 10 min, the temperature remained the same or further dropped, SSC was discontinued, and the infant was placed back under a radiant warmer or in the incubator. Similarly, if there was any bradycardia (heart rate < 100/min), desaturation (SpO_2_ < 80%), or apnea (cessation of breathing for more than 20 s or shorter if associated with bradycardia and/or desaturation) events that the infant did not recover after stimulation or by increasing the oxygen concentration, MSSC was discontinued. All episodes of MSSC within the first 7 days of life in both groups were documented. There was no specific unit policy on bathing of preterm infants during the study period.

### 2.4. Microbiological Swabs and Technique

Infant skin swabs were obtained via a non-traumatic swabbing technique using sterile cotton swab (BD EswabTM System) at three time points: (1) between 24 and 36 h (Day 2), (2) between 48 and 72 h (Day 3), and (3) on day 7 of life from the groin, axilla and umbilicus and were pooled for microbiological analyses. Maternal nasal-rectal swabs were obtained from both groups either prenatally or up to 7 days postnatally and were pooled for identification of *S. aureus* and typing, if identified. Infant mouth and rectal swabs were taken on day 7 of life and pooled for microbiological analyses and processed to identify pathogens including *enteric Gram-negative bacilli* and *S. aureus* (Table 1). The swabs were collected in tubes containing sterile normal saline (1 mL), which were initially refrigerated and processed within 12 h of receipt. An aliquot (0.1 mL) of this solution was spread (streaked evenly across the plate, manually or with an isoplater) onto a blood agar plate and allowed to dry. The blood agar plate was examined after 48 h at 37 °C. Probable pathogens (Table 1) were identified to the species level and colony counts were reported for each identified pathogen. A colony count of commensal flora/possible pathogens was performed and reported as >50 colonies of commensal flora or the number of colonies for possible pathogens.

### 2.5. Data Collection

Data were collected on maternal and infant demographics, all episodes of MSSC during the first week after birth, and the timing and results of microbiology swabs for both pre- and post-implementation groups. For the post-implementation group, we also collected the timing of and vitals during early MSSC.

### 2.6. Statistical Analysis

Statistical analyses were performed using R Version 4.4.2. Continuous variables were presented as medians with ranges for data that were not normally distributed, and categorical variables were presented as frequencies and percentages. Statistical analysis of the baseline characteristics, outcomes between the pre- and post-implementation groups and the association between maternal and infant *S. aureus* colonization in the entire cohort was conducted using the Wilcoxon rank-sum test for continuous variables and Fisher’s exact test for categorical variables, as appropriate.

## 3. Results

**Figure 1:**
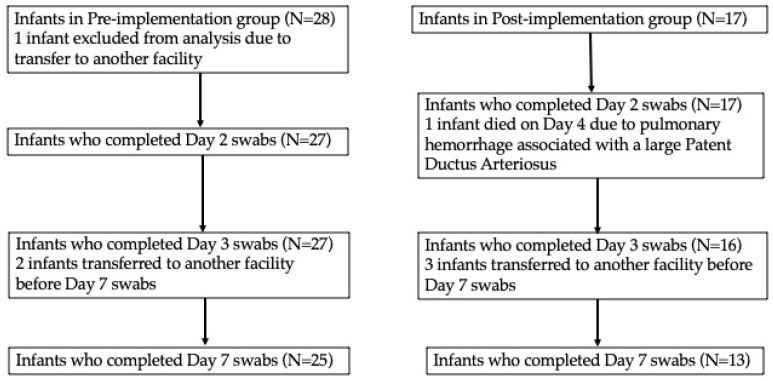
Number of infants who completed skin swabs on days 2, 3 and 7.

A total of 27 infants were included in the pre-implementation group and 17 in the post-implementation group, with 25 infants in the pre-implementation and 13 in the post-implementation group completing all the study swabs (Figure 1). Baseline characteristics were similar between the two groups, with no statistically significant differences in GA, birth weight, or delivery method (Table 2). A significantly higher proportion of infants in the pre-implementation period received empirical antibiotics at birth (26/27 (96.3%) vs. 13/17 (70.6%) *p* = 0.006) (Table 2). Infants in the post-implementation group received a significantly longer duration of SSC during the first week of life compared to the pre-implementation group (median 8.2 vs. 2.6 h, *p* = 0.03).

In the pre-implementation group, MSSC was initiated on median (range) day 2 (1–7). Early MSSC occurred within the first 24 h in 2/27 (7.4%) vs. 17/17 (100%) of the infants in the pre- and post-implementation group, respectively (*p* < 0.0001). In the post-implementation group, early MSSC occurred at a median (range) age of 13.38 (0.37–22.17) hours for a median (range) duration of 40 (30–90) min. The median (range) baseline vitals prior to MSSC were as follows: heart rate, 150 (123–181)/min; temperature, 36.8 (36.3–37.4) °C; respiratory rate, 46 (25–88)/min; SpO_2_, 96 (87–100) %; and fraction of inspired oxygen (FiO_2_), 0.21 (0.21–0.30). During MSSC, the median (range) vitals were as follows: heart rate, 142 (124–162)/min; temperature, 36.7 (36.1–36.9) °C degrees; respiratory rate, 40 (26–118)/min; oxygen saturation, 95 (88–100) %, and FiO_2_, 0.21(0.21–0.30). One out of seventeen infants developed hypothermia (lowest temperature, 36.1 °C), one developed apnea, which was resolved after gentle tactile stimulation, and one developed desaturation to 82%, which was resolved on increasing FiO_2_ from 0.21 to 0.3.

**Figure 2:**
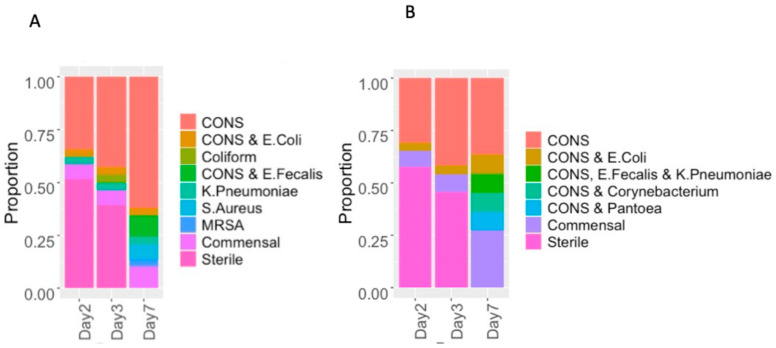
Distribution of organisms isolated on days 2, 3, and 7 in the pre-implementation (**A**) and post-implementation (**B**) groups. CONS: coagulase-negative *Staphylococci*; E. Coli: *Escherichia coli*; E. Fecalis: *Enterococcus Fecalis*; K. pneumoniae: *Klebsiella pneumoniae*; S. Aureus: *Staphylococcus aureus;* MRSA: methicillin-resistant *Staphylococcus aureus*.

There were no statistically significant differences in the colonization patterns of commensals and pathogens between the two groups at any of the time points (Table 3). Figure 2 displays the distribution of types of organisms in both the groups. The most abundant organism isolated was coagulase-negative Staphylococcus (CONS), which was present in 11/27 (40.7%), 12/27 (44.4%) and 21/25 (84%) of samples for the pre-implementation group and 9/17 (52.9%), 11/17 (64.7%) and 9/13 (69.2%) of samples for the post-implementation group, respectively, on days 2, 3, and 7. By day 7, all infants in both groups had colonized skin, with all cultures having detectable bacterial growth (Figure 2). There was no significant difference in *S. aureus* colonization rates between the two groups in either maternal nasal–rectal swabs or infant mouth–rectal swabs. Of the maternal swabs, *S. aureus* was present in 10/25 (40%) of samples for the pre-implementation group, of which 2 were methicillin-resistant *S. aureus* (MRSA) vs. 2/13 (15.4%) for the post-implementation group, among which neither were MRSA (*p* = 0.16). Amongst infant mouth–rectal swabs on day 7, *S. aureus* was present in 3/25 (12%) of samples for the pre-implementation group, 2 of which were MRSA, vs. none of the 13 for the post-implementation group (*p* = 0.57). Maternal and infant-detected *S. aureus* colonization was discordant: no maternal swabs were positive in the three children colonized with *S. aureus*, and all of the infants of mothers colonized with *S. aureus* were negative for *S. aureus* at all time points.

## 4. Discussion

In this prospective pre- and post-implementation study, we characterized the skin colonization pattern in preterm infants born between 28^0/7^ and 31^6/7^ weeks GA. Our findings suggest that early MSSC within 24 h of birth did not significantly alter the colonization pattern of commensals or pathogens. Contrary to our hypothesis and suggestions from previous studies that MSSC may alter skin colonization in preterm infants, we did not observe significant differences in colonization patterns between the two groups at any of the time points studied. By day 7, all infants in both groups had detectable bacterial growth on skin swabs.

The preterm skin differs strikingly from that of full-term infants as it is deficient in epidermal cells in a poorly defined stratum corneum and decreased epidermal thickness [14]. The stratum corneum plays an important role in maintaining skin integrity and preventing the entry of invasive pathogens [15]. Gestational age at birth influences the development of cutaneous microbiota, with lower bacterial diversity in preterm infants compared with those born at term [16,17]. The physical and functional immaturity of the skin, together with exposure to bacteria present in the immediate NICU environment and limited contact with maternal skin microorganisms predisposes the preterm skin to harboring pathogenic bacteria [17,18,19].

Previous studies on the colonization of preterm infants suggest a pattern favoring the abundance of pathogenic bacteria, with a significant impact of the NICU environment on the microbiota of neonates [17,18,20,21]. In a study of 129 hospitalized neonates, including 40 preterm infants and 89 full term infants, Younge et al. used Bayesian microbial source tracking and found an association between the infant microbiota and those found in the environment, such as objects in the immediate vicinity of the infant including the isolette, basinet, temperature probes, and vital sign monitor [17]. In a matched fecal–built environment time series analysis of fecal samples and immediate surface samples of two preterm infants, Brooks et al. demonstrated that gut colonizers such as S. epidermidis, Klebsiella pneumoniae, Bacteroides fragilis, and Escherichia coli were present abundantly in the immediate room environment, highlighting the effect of the NICU environment on infant colonization [20].

There is limited research on the effect of parental SSC on preterm skin microbiota. Hendricks-Muñoz et al. reported that in preterm infants born ≤32 weeks GA, MSSC during their NICU stay was associated with a lower prevalence of Pseudomonas, Neisseria, Proteobacteria, and Staphylococcus in saliva specimens at 1 month of age. The timing of MSSC was not reported [13]. Govindarajan et al. examined the impact of MSSC on the skin microbiome of preterm infants born <32 weeks GA and reported decreased counts of pathogens including Escherichia, S. hemolyticus, and S. hominis post MSSC [22]. MSSC was commenced after day 5; however, the day of initiation of MSSC was not reported. Neither of the studies reported the impact of MSSC on the rate of nosocomial infections.

Although skin microbiota during the first week of life in preterm infants has been studied previously [23,24], to our knowledge, this is the first study describing the effect of early MSSC within 24 h after birth on the skin microbiome. Several factors may contribute to the lack of observed differences in colonization patterns with early MSSC. First, although we aimed to eliminate the potential confounding of sepsis risk factors such as maternal GBS colonization, chorioamnionitis, and prolonged rupture of membranes, the study population consisted of relatively stable preterm infants without significant risk factors for early-onset sepsis other than prematurity. This selection criteria may have resulted in a cohort with a lower baseline risk for pathogenic colonization, potentially masking any effects of early MSSC. Second, the relatively small sample size limited the study’s power to detect differences in colonization patterns. Third, a significantly higher proportion of infants in the pre-implementation period received empirical antibiotics at birth compared to the post-implementation group (96.3% vs. 70.6%, *p* = 0.006), potentially confounding the results [16]. The lower rate of empiric antibiotic use in the post-implementation group might have been a reflection of a gradual shift in clinical practice for infants born with low risk of sepsis. However, it is likely that the process of skin colonization in preterm infants may be influenced by factors beyond early MSSC alone and may be influenced by cumulative duration of MSSC during the NICU stay and complex interactions between bacterial communities, skin immaturity and host factors.

The lack of significant difference in *S. aureus* colonization rates between the two groups and the discordance between maternal nasal–rectal swabs and infant mouth–rectal swabs on day 7 suggests that early MSSC may not substantially increase the risk of *S. aureus* transmission from mother to infant. However, the relatively low rates of *S. aureus* colonization in both groups limits the strength of conclusions that can be drawn regarding transmission risk.

Despite the lack of significant differences in colonization patterns, early MSSC was not associated with adverse outcomes or increased colonization with potential pathogens. This finding supports the safety of early MSSC in stable preterm infants and aligns with previous research demonstrating the overall benefits and safety of this practice [8]. We also demonstrated that the initiative of introducing MSSC early led to a sustained increase in MSSC throughout the first week of life. In addition to studying the effect of MSSC, our study adds to the existing literature on skin microbiota during the first week of life in preterm infants [8,24].

We acknowledge our study’s limitations, such as a small sample size and single-center design. The absence of pre-existing studies on early MSSC and skin colonization prevented us from conducting a formal sample size calculation; thus, the sample size was determined based on feasibility and convenience. We selected the gestational age group of 28^0/7^ and 31^6/7^ weeks because it represents relatively stable preterm infants suitable for studying the feasibility of early MSSC, while still being at considerable risk for developing nosocomial infections. The pre- and post-implementation design, while pragmatic, introduces the potential for confounding factors related to changes in NICU practices or environmental conditions over time. Additionally, the single-center nature of this study may limit the generalizability of the findings to other NICU settings with different patient populations or care practices. Further, we did not study the specific pattern of commensal bacteria other than CONS, Enterococcus, and Corynebacteria; however, since our primary aim was to differentiate between pathogenic and commensal organisms, this is unlikely to have significantly altered our findings.

Future studies investigating the impact of early MSSC on preterm infant colonization may benefit from employing more comprehensive microbiome analysis techniques, such as next-generation sequencing, to capture the full diversity and functional potential of the colonizing microorganisms in a larger sample and longitudinally track the evolution of the microbiome beyond the first week of life, coupled with assessments of clinical outcomes such as necrotizing enterocolitis and late-onset sepsis.

## Figures and Tables

**Table 1:** Categorization of organisms.

Pathogens	Commensal
Staphylococcus aureus β- haemolytic streptococcus groups A, B, C, G Haemophilus influenzae Streptococcus pneumoniae All aerobic Gram-negative bacilli	Coagulase-negative Staphylococcus Micrococcus species Corynebacterium species Bacillus species not B anthracis Propionibacterium species Non-pathogenic Neisseria species Enterococcus species Viridians Streptococcus group Yeasts

**Table 2:** Baseline and perinatal characteristics of the study population.

Variables	Pre-Implementation Group N = 27	Post-Implementation Group N = 17	*p*
Gestational age (weeks)	30.4 (28.0, 31.9)	30.6 (28.4, 31.9)	0.91
Female sex	15 (55.6)	7 (41)	0.54
Birthweight (grams)	1300 (540, 1880)	1370 (1010, 2300)	0.35
Multiple gestation	8 (29.6)	3 (17.6)	0.49
Maternal age (years)	33.5 (20, 43)	35 (24, 50)	0.43
Maternal infection during pregnancy	1 (3.7)	3 (17.6)	0.28
Intrapartum antibiotics	10 (37.0)	7 (41.1)	1
Duration of rupture of membranes (excluding cesarean section without labor) (hours)	1.4 (0.0, 8.3)	4.5 (0.2, 15)	0.06
Delivery by cesarean section	18 (66.7)	10 (58.8)	0.75
Apgar score 1 min	8 (2, 9)	8 (2, 9)	0.84
Apgar score 5 min	9 (4, 9)	9 (7, 9)	0.19
Antibiotics initiated at birth	26 (96.3)	12 (70.6)	**0.006**
Positive blood culture at birth	0 (0.0)	0 (0.0)	1
Skin to skin duration within first 7 days after birth (hours)	2.6 (0.0, 11.8)	8. 2 (0.0, 22.8)	**0.03**

Data are described as median (range) or number (%). *p* value in bold represents statistically significant < 0.05.

**Table 3:** Skin colonization patterns between pre- and post-implementation groups.

Skin Colonization	Pre-Implementation Group N = 27	Post-Implementation Group N = 17	*p*
Day 2	N = 27	N = 17	0.36
Commensals Pathogens Sterile	10 (37.0) 2 (7.4) 15 (55.6)	10 (58.8) 1 (5.9) 6 (35.3)	
Day 3	N = 27	N = 16	0.24
Commensals Pathogens Sterile	13 (48.1) 3 (11.1) 11 (40.7)	12 (75.0) 1 (6.3) 3 (18.8)	
Day 7	N = 25	N = 13	0.22
Commensals Pathogens Sterile	21 (84.0) 4 (16.0) 0 (0.0)	8 (61.5) 5 (38.5) 0 (0.0)	
Staphylococcus aureus on maternal nasal–rectal swabs	N = 25	N = 13	0.16
	10 (40.0)	2 (15.4)	
Staphylococcus aureus in infant mouth–rectal swabs on day 7 of life	N = 25 4 (16.0)	N = 13 0 (0.0)	0.54

Data are presented as number (%).

## Data Availability

Data supporting reported results are available upon request and are housed on a secured server with the study’s authors P.D. and V.S.
